# Medication management support in diabetes: a systematic assessment of diabetes self-management apps

**DOI:** 10.1186/s12916-019-1362-1

**Published:** 2019-07-17

**Authors:** Zhilian Huang, Elaine Lum, Geronimo Jimenez, Monika Semwal, Peter Sloot, Josip Car

**Affiliations:** 1 Centre for Population Health Sciences, Lee Kong Chian School of Medicine, Nanyang Technological University, Clinical Sciences Building, Level 18, 11 Mandalay Road, Singapore, 308232 Singapore; 2 NTU Institute for Health Technologies (HealthTech NTU), Interdisciplinary Disciplinary School, Nanyang Technological University, Singapore, Singapore; 3 Institute of Health and Biomedical Innovation, Queensland University of Technology, Brisbane, Australia; 4 School of Clinical Sciences, Faculty of Health, Queensland University of Technology, Brisbane, Australia; 5 Institute for Advanced Study, University of Amsterdam, Amsterdam, The Netherlands; 6 ITMO University, Saint Petersburg, Russia; 7 Complexity Institute, Nanyang Technological University, Singapore, Singapore

**Keywords:** Health apps, Digital health, Diabetes, Medication adherence, Evidence-based guidance

## Abstract

**Background:**

Smartphone apps are becoming increasingly popular for supporting diabetes self-management. A key aspect of diabetes self-management is appropriate medication-taking. This study aims to systematically assess and characterise the medication management features in diabetes self-management apps and their congruence with best-practice evidence-based criteria.

**Methods:**

The Google Play and Apple app stores were searched in June 2018 using diabetes-related terms in the English language. Apps with both medication and blood glucose management features were downloaded and evaluated against assessment criteria derived from international medication management and diabetes guidelines.

**Results:**

Our search yielded 3369 Android and 1799 iOS potentially relevant apps; of which, 143 apps (81 Android, 62 iOS) met inclusion criteria and were downloaded and assessed. Over half 58.0% (83/143) of the apps had a medication reminder feature; 16.8% (24/143) had a feature to review medication adherence; 39.9% (57/143) allowed entry of medication-taking instructions; 5.6% (8/143) provided information about medication; and 4.2% (6/143) displayed motivational messages to encourage medication-taking. Only two apps prompted users on the use of complementary medicine. Issues such as limited medication logging capacity, faulty reminder features, unclear medication adherence assessment, and visually distracting excessive advertising were observed during app assessments.

**Conclusions:**

A large proportion of diabetes self-management apps lacked features for enhancing medication adherence and safety. More emphasis should be given to the design of medication management features in diabetes apps to improve their alignment to evidence-based best practice.

**Electronic supplementary material:**

The online version of this article (10.1186/s12916-019-1362-1) contains supplementary material, which is available to authorized users.

## Background

Medication adherence, broadly understood as the act of taking medicines as prescribed by the healthcare provider, is important for achieving treatment goals [1]. This is paramount for chronic conditions such as diabetes. However, studies have shown that approximately 33% of oral medications and 38% of insulin for type 2 diabetes (T2D) are not taken/used as prescribed [2, 3] due to forgetfulness, inconvenience, negative treatment beliefs, fear of injections and a myriad of other personal and health system factors [4].

Medication management strategies have been developed and implemented to assist people in adhering to their medications. These strategies include education on disease management, simplification of dosing regimen, counselling, reminders, or a combination of these methods [5, 6]. Digital solutions have also been studied in the past 20 years to assist in medication adherence. Although research has shown that mobile text messaging can double the odds of medication adherence in chronic diseases [7], more successful interventions often involved the use of two-way communication [8] and were tailored to individual needs [9, 10]. This suggests the need for innovation and a combination of measures that go beyond basic reminders to improve medication adherence.

Smartphone apps have gained popularity in diabetes self-management in recent years. Compared to SMS reminders, smartphone apps have the advantage of performing more sophisticated medication management functions such as pill organisation, tracking of medication-taking, information provision, and adherence assessment [6]. With the rise in the number of smartphone users [11, 12] and integration of smartphone apps in daily living [13], a myriad of apps were developed to assist people in adhering to their medications. Despite the large number (approximately 400) of accessible and free apps for medication self-management in the app market in recent years [14], the majority of these apps lacked useful, desirable features for medication adherence [15]. According to national digital health consumer surveys, only 11% of respondents tracking health goals tracked their medications [16]. Medication adherence is also least likely to be tracked in an app (10%) amongst other trackable health-related metrics like physical activity, heart rate and sleep [17].

Currently, the large number of available diabetes management apps provides an opportunity to support medication management, but also represents missed opportunities to improve care for people with diabetes with gaps that fall short of users’ needs [18]. It is unclear if diabetes apps are adequately incorporating medication management strategies and if app features are aligned with best-practice evidence-based recommendations [19]. Improvements in app quality and utility can only be realised if gaps in app features are identified. We constructed a diagram linking good medication management practice with possible app features and systematically assessed and characterised the medication management features in available apps for T2D self-management. We discussed the implications of our findings in relation to diabetes management and provided suggestions to address the identified gaps.

## Methods

### Development of app assessment criteria

**Fig. 1:**
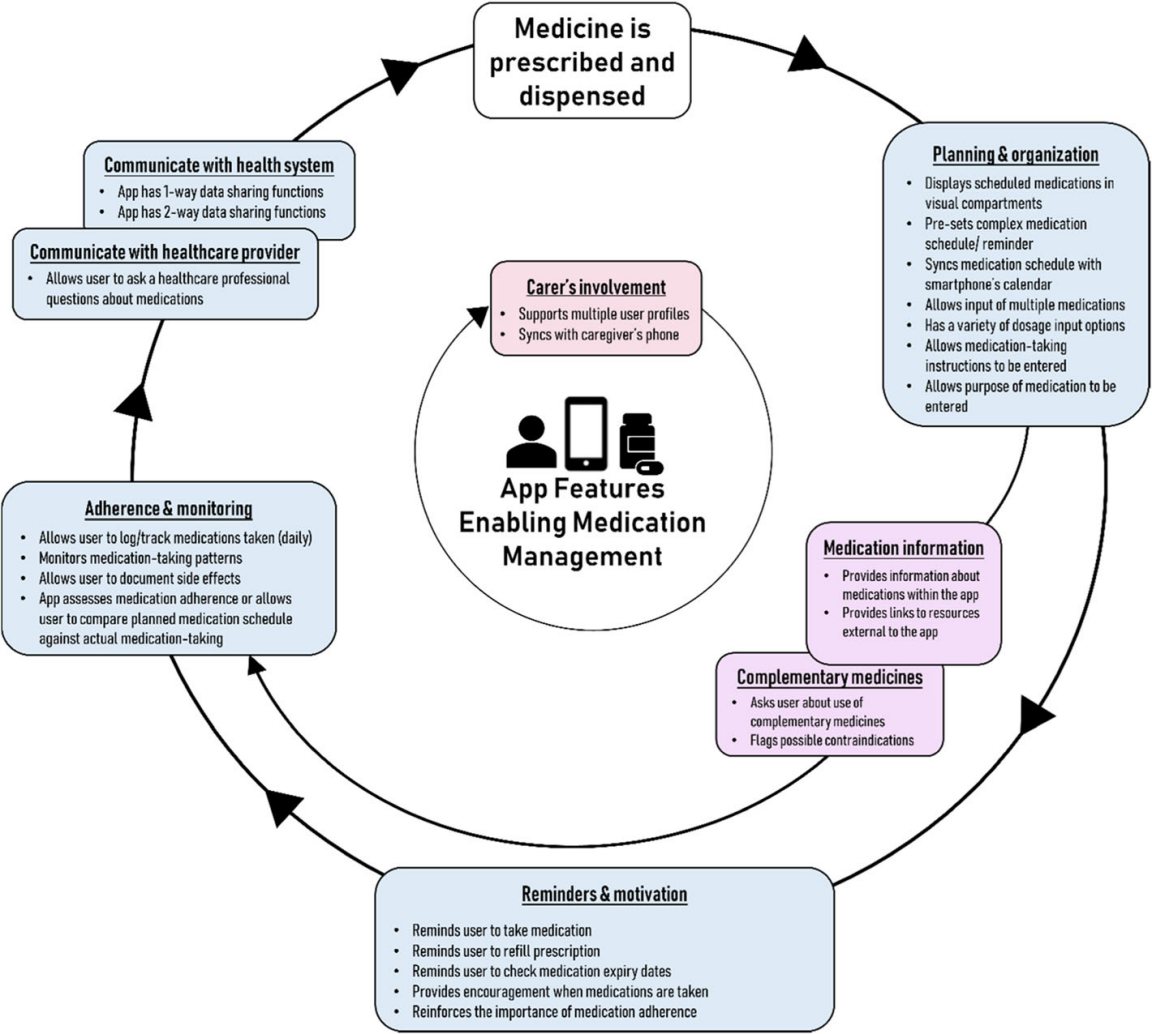
Diagram of app features mapped with factors for good medication management practice. Statements from international medication management guidelines and literature were grouped according to adaptations from Stowasser’s medicines management pathway. App features were then mapped with the groupings to link the features into a medication management pathway. The mapped features were used to develop evidenced-based criteria for app assessment. Different box colours were used to differentiate layers of the medication management app pathway

Statements from international medication management guidelines and literature were extracted based on their applicability to chronic disease self-management [20–29]. Similar concepts (i.e. factors) were grouped, mapped with possible app features and assigned a group heading for classification purposes. We then linked the groups of app features by adapting the diagram from Stowasser’s medication management pathway [30]. Figure 1 illustrates the relationship between the factors for good medication management practice and possible app features.

The possible app features for diabetes self-management (from Fig. 1) were developed into app assessment criteria (Table 1). Each assessment criterion was mapped back to medication management guidelines. For example, the assessment criteria “The app allows users to assess medication adherence by comparing planned and actual medication taking”, operationalised through the app’s logging and tracking features, were mapped to guidelines recommending clinicians to “Routinely assess adherence during prescribing, dispensing, and reviewing medicines” for medication adherence. All app assessment criteria had binary responses (Yes/No) for consistency.

**Table 1:** App assessment criteria with the corresponding guidance/evidence extracted from international medication management guidelines and literature

S/N	App assessment criteria	Excerpt of extracted guidance/justification	References supporting the guidance
1	Planning and organisation		
1.1	The app has a feature that allows the user to display scheduled medications as different visual compartments (e.g. visual pillbox in the app)	Reduce dosing complexity: Use blister or compartmentalised boxes to reduce dosing complexities	[21, 24, 25, 28]
1.2	The app has a feature that allows the user to switch between daily and weekly medication schedule displays		
1.3	The app has a feature that allows the user to schedule medication-taking on alternate days (e.g. Pill A on Monday, Wednesday, Friday; Pill B on Tuesday, Thursday, Saturday)		
1.4	The app has a feature to enter the purpose of the medication	Planning and organisation: Develop an individualised, documented self-management plan including the plan’s start and review date, conditions being managed, description of the medication (frequency, dose, strength, instructions, known reactions, and allergies, length of treatment)	[20, 21, 24, 25, 28]
1.5	The app has a feature that allows the user to enter special instructions for medication (e.g. taken before food)		
1.6	The app has a feature that allows the user to organise “take as needed” medications in a separate section from medicines with a fixed regimen		
1.7	The app has a feature that allows the user to enter/log at least 4 different medications at any given time		
1.8	The app has a variety of dosage input options (e.g. subcutaneous insulin for diabetes, oral medications)		
1.9	The app has a feature that allows the user to document allergies (i.e. via prompts/greyed out instructions or a separate tab)		
1.10	The app has a feature that allows the user to sync medication-taking schedule with the phone calendar	For user’s convenience without having to open a separate calendar to view medication schedule.	
2	Adherence and monitoring		
2.1	The app has a feature that allows the user to record the fraction of an actual pill or volume of a liquid medication prescribed (i.e. ½ pill or 5 ml of a syrup) to be recorded.	Oral tablets may be prescribed as a fraction of one and liquids are prescribed as a specified volume. Apps that do not allow this will be less helpful and could introduce errors.	
2.2	The app has a feature that allows users to document medication-intake	Monitoring: Record medicines taken, self-monitor the condition and report all adverse reactions.	[20, 21, 24, 25, 28]
2.3	The app has a feature that allows users to record notes on any medication event (i.e. a “note/comment” section at the logging page or a as a separate tab)		
2.4	The app has a feature that allows users to document medication side-effects (i.e. via prompts/greyed-out instructions or a separate tab)		
2.5	The app has a feature that assesses medication adherence by comparing planned and actual medication taking (E.g. the app generates weekly percentage of adherence or has a visual display).	Adherence assessment: Routinely assess adherence during prescribing, dispensing, and reviewing medicines.	[21, 24, 25, 28, 29]
3	Information provision		
3.1	The app has a feature that provides users with information about the prescribed medication	Information provision: Repeatedly offer clear, understandable, and relevant information about the medication prescribed. Provide resources to where information about medication can be obtained.	[20, 23, 24, 28]
3.2	The app has a feature that provides users with resources (in-app or external link) to access information about the prescribed medication		
4	Complementary medicines		
4.1	The app has a feature that asks users about the use of complementary medicines	Complementary medicine: Take into account all complementary medicines the person is taking or using, and its purpose.	[20–23, 27]
4.2	The app has a feature that flags possible contraindications with the use of complementary medicines		
5	Reminders		
5.1	The app has a feature that allows users to set up reminders for taking medications	Reminders: Reminders have shown to improve adherence to medicines despite inconclusive evidence.	[20, 23, 24, 28]
5.2	The app has a feature that allows users to set up reminders to refill prescriptions	Refill medication: Prescription refill is an indirect method for measuring medication adherence, and could alert prescribers and pharmacists to problems of adherence.	
6	Motivation		
6.1	The app has a feature that provides statements to motivate users about the importance of medication adherence	Behavioural change: Positive reinforcements are important in sustaining behavioural change (Guidelines). Providing consequences and benefits of effective medication adherence helps the patient to understand the need and to establish motivation to adhere to medication [26]. Positive reinforcements are important in sustaining behavioural change [27].	[21, 29]
6.2	The app has a feature that provides encouragement when medication is taken on schedule (i.e. encouraging messages, “badges or awards”)		
7	Caregiver’s involvement		
7.1	The app has a feature that allows users to sync medication-taking schedule with caregiver’s phone	Carer’s involvement: Keep an up-to-date list of all medicines the patient is taking and take note of any allergic or adverse reactions to medicines.	[21, 24, 25, 28]
7.2	The app has a feature that supports multiple user profiles (e.g. For family members or carers)		
8	Communication with healthcare provider		
8.1	The app has a feature that allows users to contact a healthcare provider regarding queries on medication	Communication with health provider: Establish the most effective way of communicating with each patient.	[21, 24, 25, 28, 29]
9	Communication with health system		
9.1	The app has a feature that supports data export	Communication within/across health settings: Health and social care practitioners should share relevant information about the person and their medicines when a person transfers from one care setting to another. Use the most effective and secured way with one or multiple approaches, such as secure electronic communication.	[20, 21, 24, 25, 28]

Legend: *CG76*: Medicines adherence: involving patients in decisions about prescribed medicines and supporting adherence [25]; *NG5:* Medicines optimisation: the safe and effective use of medicines to enable the best possible outcomes [20]; *NCCPC:* Clinical guidelines and evidence review for medicines adherence: involving patients in decisions about prescribed medicines and supporting adherence [Full guideline and evidence] [21]; *King’s fund:* Polypharmacy and medicines optimisation: Making it safe and sound [24]; *AHRQ* [29] (Evidence Report); *APAC*: Australian Pharmaceutical Advisory Council Guiding principles for medication management in the community [28]

### App selection and assessment

#### Search strategy

The app search and selection methods in this study were adapted from principles of a systematic review to ensure minimisation of bias. Diabetes terms were searched to capture apps that were marketed for diabetes self-management. The Google Play and Apple app stores were searched in June 2018 via an app market explorer (https://42matters.com/) which covers both app stores in 55 countries. The search terms used in the English language were “(Diabetes OR Diabetic OR Diabetics) OR (glucose OR glycaemic OR glycemic OR blood sugar OR HbA1c OR A1c) OR insulin”, which produced a list of app titles and descriptions for screening.

#### Screening

App titles and descriptions were screened for inclusion and exclusion using the following process: a random sample of 100 apps was first screened by two researchers to ensure consistency in the interpretation of the inclusion and exclusion criteria. Differences in interpretation were resolved via consensus discussion. Unclear titles and descriptions were conservatively included for downloading and re-screening until an inter-rater agreement of above 80% was achieved. Apps available on both the iOS and Android platforms were treated as unique apps due to possible differences in versions across platforms.

The following inclusion and exclusion criteria were used:

Inclusion criteria:

- Apps with medication self-management features
- Apps with any blood glucose logging features
- Apps in the English language
- Free apps and apps requiring payment

Exclusion criteria:

- Patient health portals linking to patients’ electronic health records
- Apps that were not updated after January 1, 2017
- Intended for healthcare professionals
- Insulin calculators/bolus correctors only
- Apps with exclusive blood glucose monitoring device tie-in requirement
- Apps duplicated on the same platform
- Apps with regional restrictions
- Technical problems (e.g. crashes, screen hangs, unable to login, unable to download)

#### App assessments

The medication management features of selected apps were evaluated against the app assessment criteria. Three apps with extensive features were selected to pilot app assessment and refine the assessment criteria. Team members underwent a calibration exercise to ensure consistency in interpretations. All selected apps were then split among six researchers for assessment (see Additional file 1 for the list of smartphones and their OS system). App developers were contacted for access to restricted apps that were free to download. Apps that could not be accessed within a month of contact were excluded from the study. Free apps which offered additional features upon payment were evaluated with the additional features in place.

### Statistical analysis

#### Screening

Cohen’s kappa was used to calculate the inter-rater agreement between two researchers at the screening process. An agreement score of between 0.6 and 0.8 represents a reasonably good agreement between the reviewers [31]; a 0.8 cut-off score was used in this study due to the broad inclusion criteria.

#### App assessments

Apps were grouped by platform (i.e. Android, iOS) and profiled according to its features (i.e. reminders, tracking, monitoring) using descriptive statistics. Pearson’s chi-squared test was used for comparisons between groups. A two-tailed Fisher’s exact test was used where the expected count is less than five in a group. Statistical significance was set at *p* value < 0.05. All analyses were performed using SPSS version 22 [32].

## Results

**Fig. 2:**
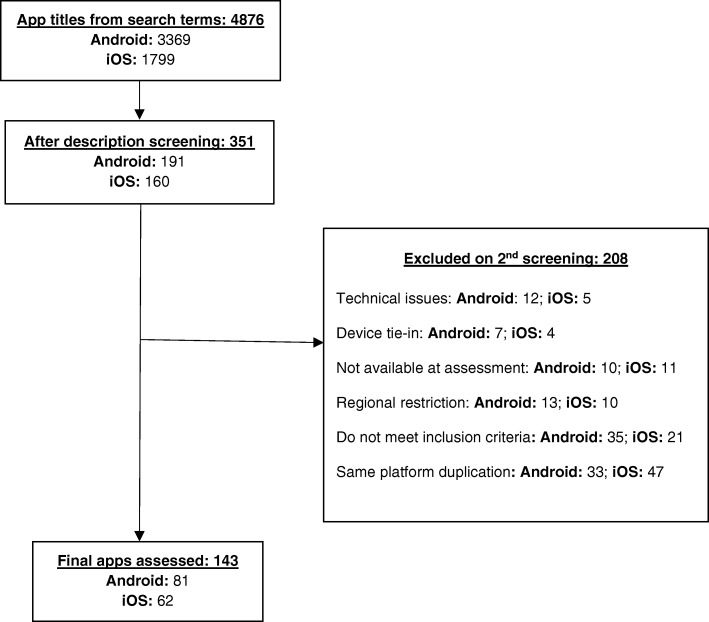
Flowchart for app selection. The search terms “(Diabetes OR Diabetic OR Diabetics) OR (glucose OR glycaemic OR glycemic OR blood sugar OR HbA1c OR A1c) OR insulin” yielded 4876 results from (https://42matters.com). After screening the app descriptions for relevance, 351 apps were downloaded for assessment; of which, 208 apps were excluded due to (i) technical issues, (ii) device tie-in, (iii) not available at app assessment, (iv) regional restriction, (v) do not meet inclusion criteria on second screening and (vi) duplication of apps on the same platform. Finally, 143 apps (81 Android; 62 iOS) were fully assessed against the app assessment criteria in Table 1

The search terms yielded 3369 Android and 1799 iOS apps. After title and description screening, 191 (5.7%) Android and 160 (8.9%) iOS apps remained for downloading. Apps were further excluded due to their unavailability, exclusive device tie-in requirement, technical issues, not meeting the inclusion criteria (i.e. non-English), and if they were duplicated on the same platform. Restricted apps that received no reply from developers were also excluded from the study. Finally, 143 apps (81 Android, 62 iOS) were downloaded and assessed against the app assessment criteria (Fig. 2).

The search terms “(Diabetes OR Diabetic OR Diabetics) OR (glucose OR glycaemic OR glycemic OR blood sugar OR HbA1c OR A1c) OR insulin” yielded 4876 results from (https://42matters.com). After screening the app descriptions for relevance, 351 apps were downloaded for assessment; of which, 208 apps were excluded due to (i) technical issues, (ii) device tie-in, (iii) not available at app assessment, (iv) regional restriction, (v) do not meet inclusion criteria on second screening, and (vi) duplication of apps on the same platform. Finally, 143 apps (81 Android; 62 iOS) were fully assessed against the app assessment criteria in Table 1.

### Characteristics of included apps

The frequency of app features grouped by platform is shown in Table 2.

**Table 2:** Frequency of app features grouped by platform

Classification		App features	All apps (*n* = 143) (%)	Android (*n* = 81) (%)	iOS (*n* = 62) (%)	*p* value
Planning and organisation	1.1	The app has a feature that allows the user to display scheduled medications as different visual compartments (e.g. visual pillbox in the app)	7 (4.9)	4 (4.9)	3 (4.8)	1.000^
	1.2	The app has a feature that allows the user to switch between daily and weekly medication schedule displays	4 (2.8)	3 (3.7)	1 (1.6)	0.633^
	1.3	The app has a feature that allows the user to schedule medication-taking on alternate days (e.g. Pill A on Monday, Wednesday, Friday; Pill B on Tuesday, Thursday, Saturday)	37 (25.9)	24 (29.6)	13 (21.0)	0.241
	1.4	The app has a feature to enter the purpose of the medication	42 (29.4)	22 (27.2)	20 (32.3)	0.507
	1.5	The app has a feature that allows the user to enter special instructions for medication (e.g. taken before food)	57 (39.9)	33 (40.7)	24 (38.7)	0.806
	1.6	The app has a feature that allows the user to organise “take as needed” medications in a separate section from medicines with a fixed regimen	12 (8.4)	6 (7.4)	6 (9.7)	0.628
	1.7	The app has a feature that allows the user to enter/log at least 4 different medications at any given time	98 (68.5)	56 (69.1)	42 (67.7)	0.859
	1.8	The app has a variety of dosage input options (e.g. subcutaneous insulin for diabetes, oral medications)	123 (86.0)	66 (81.5)	57 (91.9)	0.074
	1.9	The app has a feature that allows the user to document allergies (i.e. via prompts/greyed out instructions or a separate tab)	5 (3.5)	4 (4.9)	1 (1.6)	0.389^
	1.10	The app has a feature that allows the user to sync medication-taking schedule with the phone calendar	7 (4.9)	2 (2.5)	5 (8.1)	0.239^
Monitoring and adherence	2.1	The app has a feature that allows the user to record the fraction of an actual pill or volume of a liquid medication prescribed (i.e. ½ pill or 5 ml of syrup) to be recorded.	83 (58.0)	45 (55.6)	38 (61.3)	0.491
	2.2	The app has a feature that allows users to document medication-intake	112 (78.3)	57 (70.4)	55 (88.7)	0.008*
	2.3	The app has a feature that allows users to record notes on any medication event (i.e. a “note/comment” section at the logging page or as a separate tab)	70 (49.0)	35 (43.2)	35 (56.5)	0.116
	2.4	The app has a feature that allows users to document medication side-effects (i.e. via prompts/greyed-out instructions or a separate tab)	5 (3.5)	4 (4.9)	1 (1.6)	0.389^
	2.5	The app has a feature that assesses medication adherence by comparing planned and actual medication taking (e.g. the app generates weekly percentage of adherence or has a visual display).	24 (16.8)	17 (21.0)	7 (11.3)	0.175
Information provision	3.1	The app has a feature that provides users with information about the prescribed medication	12 (8.4)	6 (7.4)	6 (9.7)	0.628
	3.2	The app has a feature that provides users with resources (in-app or external link) to access information about the prescribed medication	8 (5.6)	4 (4.9)	4 (6.5)	0.727^
Complementary medicines	4.1	The app has a feature that asks users about the use of complementary medicines	2 (1.4)	0 (0.0)	2 (3.2)	0.186^
	4.2	The app has a feature that flags possible contraindications with the use of complementary medicines	0 (0.0)	0 (0.0)	0 (0.0)	1.000^
Reminders	5.1	The app has a feature that allows users to set up reminders for taking medications	83 (58.0)	47 (58.0)	36 (58.1)	0.996
	5.2	The app has a feature that allows users to set up reminders to refill prescriptions	9 (11.1)	6 (9.7)	15 (10.5)	0.782
Motivation	6.1	The app has a feature that provides statements to motivate users about the importance of medication adherence	8 (5.6)	3 (3.7)	5 (8.1)	0.293^
	6.2	The app has a feature that provides encouragement when medication is taken on schedule (i.e. encouraging messages, “badges or awards”)	6 (4.2)	3 (3.7)	3 (4.8)	1.000^
Caregiver’s involvement	7.1	The app has a feature that allows users to sync medication-taking schedule with caregiver’s phone	9 (6.3)	5 (6.2)	4 (6.5)	1.000^
	7.2	The app has a feature that supports multiple user profiles (e.g. For family members or carers)	22 (15.4)	11 (13.6)	11 (17.7)	0.494
Communication with healthcare provider	8.1	The app has a feature that allows users to contact a healthcare provider regarding queries on medication	16 (11.2)	7 (8.6)	9 (14.5)	0.269
Communication with health system	9.1	The app has a feature that supports data export	89 (62.7)	44 (55.0)	45 (72.6)	0.036*

^Two-tailed *p* value calculated using Fisher’s exact test as the expected count is less than 5 in at least a group

*Statistical significance *p* < 0.05 in the comparison between Android and iOS app features

#### Planning and organisation

More than two thirds of the apps allowed users to input insulin doses (86.0%, 123/143) and record multiple medications (68.5%, 98/143), while less than half of the apps allowed users to input special instructions (39.9%, 57/143) or the purpose (29.4%, 42/143) of the medication. A low proportion of the apps supported users in managing dosing complexities with digital visual compartments (4.9%, 7/143), toggling between daily and weekly displays (2.8%, 4/143), and pre-setting complex medication schedules (25.9%, 37/143). Few apps also specifically asked the user to document allergies (3.5%, 5/143) or allowed the user to sync medication schedules with the smartphone’s calendar (4.9%, 7/143). No differences in app features were observed between the operating platforms.

#### Monitoring and adherence

A significantly higher proportion of iOS apps had a basic medication tracking feature (Android 70.4%, 57/81; iOS 88.7%, 55/62; *p* = 0.008) compared with Android apps. Although the note-taking feature of the apps (49.0%, 70/143) allowed free text entry, very few specifically asked the user to document medication side-effects (3.5%, 5/143). More than half of the apps allowed recording of dose fractions (58%, 83/143), but 16.8% (24/143) allowed the user to review medication adherence by comparing planned and actual medication taking.

#### Medication information

Few apps provided in-app medication information (8.4%, 12/143) or external resources to medication information (5.6, 8/143%). Two iOS apps prompted the user on the use of complementary medicine, but none of these apps were able to flag possible contraindications with the use of complementary medicines.

#### Reminders, motivation and caregiver’s involvement

Just over half of the apps (58.0%, 83/143) had a medication reminder feature. Few apps were able to remind the user to refill their medication (11.1%, 9/143), reinforced the importance of medication adherence (5.6%, 8/143) or encouraged medication-taking as scheduled with motivational messages (4.2%, 6/143). There were also very few apps that supported caregiver’s involvement, such as supporting multiple user profiles (15.4%, 22/143) or enabling data syncing with a caregiver’s phone (6.3%, 9/143).

#### Communication with provider and health system

Few apps allowed the user to ask a health professional questions about medications (11.2%, 16/143). For one-way data sharing, a significantly higher proportion of iOS apps had data export features (Android 55.0%, 44/81; iOS 72.6%, 45/62; *p* = 0.036).

#### Additional findings

We further compared Android apps with < 100,000 downloads against those with higher downloads (≥ 100,000) (Additional file 2). Although only a small number (17/81) of apps were downloaded ≥ 100,000 times, a significantly higher proportion of these apps have features that allowed the user to separate medications into “take as needed” sections, document medication-intake, vary dosage input options, set up reminders to refill prescriptions, sync medication-taking schedule with caregiver’s phone, support multiple user profiles and support data export. We were unable to analyse iOS apps in the same manner as the number of downloads was not available from the Apple app store.

Several additional issues were found during the app assessments. First, the medication logging feature of some apps was limited by the absence of features such as timestamp, dosage, measurement unit and medication label. For example, one app restricted oral medications input to a maximum dosage of 99.9 mg despite the much higher dosage of some diabetes oral medications. Another app limited medication label to “medication 1” and “medication 2”. A few other apps did not allow medication logging to be retrospective nor allowed the user to tag an event (i.e. physical activity or a meal) to the medication. Second, some apps had reminder features which did not allow the user to set a recurring alarm nor pre-set a medication-taking schedule. In addition, a few apps had hard-to-find reminder features, faulty alarms that did not work or could not be stopped and delayed notifications. Lastly, we observed one app with inaccurate adherence tracking. The app divided the percentage of medication “taken” and “skipped” in a pie chart without differentiating the type of medication nor the time period of the entry. Other issues include visually distracting advertisements (subjective to assessor’s judgement), inability to set up a personal account, poor user interface and functional errors during usage (i.e. crashes).

## Discussion

We identified, downloaded and systematically evaluated 143 apps against assessment criteria derived from international medication management guidelines. There were few differences in app features between Android and iOS apps except for a higher proportion of iOS apps having medication-intake documentation and data export features. Most of the assessed apps, including apps with higher downloads (≥ 100,000), have basic logging and tracking features for diabetes medication, but lacked features that could enhance medication adherence and safety. We identified the following gaps in the assessed apps. First, many diabetes apps lacked any form of medication management features, which concurs with a 2017 study which found that only 50% of the highest rated iOS diabetes management apps had medication adherence features [33]. A separate study by our team which employed a similar search strategy also found that only 43% of the accessible diabetes self-management apps had medication management features [34]. This may be attributed to a lack of emphasis given to medication adherence in diabetes management. Amongst the assessed apps with medication management features, a large proportion did not have important features such as a basic reminder feature, the capability to enter medication-taking instructions and medication adherence review. Apps devoid of essential features for enhancing medication adherence are less likely to be useful in helping users adhere to their medications.

Second, less than 10% of the apps provided any information on diabetes medication or allowed the user to communicate with a healthcare professional. Although this feature is more important for users needing to adjust to a new medication therapy, having information on medication will be beneficial as diabetes is likely to progress over time. Third, only two iOS apps prompted the user if complementary medicine was being used. The use of complementary medicine is common in many cultures and can lead to contraindications [35]. Stopping conventional medication in favour of complementary medicine can also lead to ineffective or adverse treatment outcomes. It would be important for app developers to include a cautionary message or features to alert users to potential contraindications. Documentation of allergies is also important to flag possible medication or food-related contraindication but only 3.5% of the assessed apps have this capability.

Fourth, less than 5% of the apps had features that provided any form of motivation to the user. A few apps that encouraged medication adherence had interactive features that could possibly increase the time spent on the app. Sustained app use may increase medication-taking awareness of users who may otherwise not remember to take their medications. Lastly, about 40% of the apps do not allow data export, which can assist the individual or a healthcare provider to review treatment plans and goals.

An explanation for the lack of evidence-based features in health apps may be in the absence of healthcare providers’ involvement in the development of the app [19]. Only 13.6% of the apps for medication adherence were developed with the involvement of a healthcare provider and only 1% of the apps were evidence-based according to another study [14]. Intermittent app use or app intervention failure may sometimes be caused by a lack of useful app features rather than apps being ineffective per se. Many medication adherence app intervention studies focused on reminding the user to take their medications, but the quality of reminder features and alignment with evidence-based recommendations were unclear [36, 37]. Assessing the app against a medication management checklist (as illustrated in Fig. 1) before the intervention will better align the app for its purpose.

While our study focused on users with T2D, 86% of the assessed apps allow users to log and track insulin doses and hence can also be used by people with type 1 diabetes (T1D). However, adherence to insulin is more challenging than to oral medications due to barriers such as fear of injections, embarrassment of injecting in public, concerns over cost and side effects such as hypoglycaemia) [3, 38, 39]. These barriers cannot be overcome solely by the use of a medication management app, although apps could potentially support adherence to insulin therapy via incorporating patient education.

Strengths of this study include that our app assessment criteria were developed referring to evidence-based guidelines and covered a broader scope on medication management compared to other studies [6, 15]. We also assessed free apps and apps requiring payment which were not limited to one country’s app store.

Despite attempts to minimise bias, there were limitations to the study. We were unable to cover the entire spectrum of medication management apps. Instead, we chose to focus on diabetes due to its prevalence and the need for long-term medication management. The assessment may also not reflect the current state of the apps due to constant updates. However, we attempted to cover all apps at a particular time point and believe that our findings remain unchanged as a previous study showed that the quality of apps in terms of alignment with evidence-based guidelines did not improve within a 2-year period [40]. The app assessment criteria for this study were selected based on their perceived usefulness to people with T2D requiring long-term medication management. Other criteria such as focusing on shared decision-making when medications are not taken as intended could be derived for future assessments. Additionally, we did not investigate the app’s ability to flag medication contraindications nor assessed the content of the medication information provided in these apps. Although the app assessment criteria were developed from the perspective of chronic disease management, we believe we covered the app features important for medication management in diabetes. Since diabetes self-management requires additional self-care activities such as blood glucose monitoring, physical activity and diet modifications, other features of the app should be considered in assessing the overall quality of the app.

A minimum standard (i.e. certification or selection of apps using an evidence-based checklist) could be one way to raise the standard of medication management features of apps. The implementation of the NHS digital library and the FDA “precertification” programme for mobile apps are precedents of tools to objectively evaluate apps, although gaps still exist in the app marketplace in meeting patients’ and clinicians’ needs [41]. We also believe that app stores should play a greater role in quality assurance of health/medical apps. In addition to certification, health app developers should take active steps to ensure that their apps meet minimum standards by co-designing apps with potential users and by continuing to upgrade their app. Healthcare providers can take a more active role in participating in app co-design and work with their patients to effectively use an app to manage chronic conditions. Researchers planning for medication adherence app intervention studies should also be aware of the shortcomings of current apps when evaluating the effectiveness of these apps in improving medication adherence.

The list of assessment criteria is non-exhaustive and should be tailored to patient needs and advancements in technology. Given that these assessment criteria may be applicable to medication management of other chronic diseases, future studies can explore apps specific to other chronic diseases to determine if similar gaps exist. Studies can also explore the usability of these apps for better patient experience, and the efficacy of the medication management features in improving medication adherence in different settings.

## Conclusions

Our systematic, broad and evidence-based assessment of smartphone apps provides an overview of medication management features of diabetes self-management apps. A large proportion of the apps lacked features that were useful for enhancing medication adherence and safety, such as the capability to enter allergies and medication-taking instructions, functional reminders, information provision and prompts for the usage of complementary medicine. These gaps represent missed opportunities for better app features which can potentially enhance digital medication management in people with T2D. More emphasis should be given to the inclusion and design of medication management features in diabetes apps. Healthcare providers, app developers and researchers should be involved in the co-design of health apps in order to improve their quality and be aware of the shortcomings of current apps when making recommendations about their effectiveness.

## Additional files

Additional file 1: List of smartphones and their operating systems used for app assessment. (DOCX 15 kb)

Additional file 2: Frequency of app features grouped by number of downloads (Android apps only). (DOCX 26 kb)

## Data Availability

The datasets used and/or analysed during the current study are available from the corresponding author on reasonable request.

## Abbreviations

T1D: Type 1 diabetes
T2D: Type 2 diabetes
