# Supplementary material for: Medication management support in diabetes: a systematic assessment of diabetes self-management apps
Source: BMC Med. 2019 Jul 17;17:127. doi: 10.1186/s12916-019-1362-1 (PMC6636047; doi:10.1186/s12916-019-1362-1)
Supplement: Supplementary file 1 — List of smartphones and their operating systems used for app assessment. (DOCX 15 kb) [file 12916_2019_1362_MOESM1_ESM.docx]

**Additional file 1**

**List of smartphones and their operating systems used for app assessment**

| **Phone model** | **Original Operating system** | **Operating system version*** |
| --- | --- | --- |
| iPhone 5c | iOS | iOS 10.3.3 |
| iPhone 6 | iOS 8.0 | iOS 11.1 |
| iPhone7 | iOS 10.0.1 | iOS 11.1 |
| Samsung Galaxy A5 | Android 6.0.1 "Marshmallow" | Android 7.0 (Nougat) |
| Samsung Galaxy A7 | Android 6.0.1 "Marshmallow" | Android 7.0 (Nougat) |
| Samsung J7 Pro | Android 7.0 (Nougat) | Android 7.0 (Nougat) |
| Samsung Galaxy Note 4 | Android 4.4.4 “KitKat” | Android 6.0.1 "Marshmallow" |
| OnePlus 3T | OxygenOS | OxygenOS |

*Listed above are the phones and OS versions we used for our app assessment. We listed the earliest OS version at the start of the apps assessment; minor OS version updates may occur during the few months of app assessment but they did not affect the apps .
