# Supplementary material for: Medication management support in diabetes: a systematic assessment of diabetes self-management apps
Source: BMC Med. 2019 Jul 17;17:127. doi: 10.1186/s12916-019-1362-1 (PMC6636047; doi:10.1186/s12916-019-1362-1)
Supplement: Supplementary file 2 — Frequency of app features grouped by number of downloads (Android apps only). (DOCX 26 kb) [file 12916_2019_1362_MOESM2_ESM.docx]

**Additional file 2**

Frequency of app features grouped by number of downloads (Android apps only)

| **Classification** | **S/N** | **App features** | **All apps (n = 81) (%)** | **<100,000 downloads (n = 64) (%)** | **≥100,000 downloads (n = 17) (%)** | **p-value** |
| --- | --- | --- | --- | --- | --- | --- |
| Planning and Organisation | 1.1 | The app has a feature that allows the user to display scheduled medications as different visual compartments (e.g. visual pillbox in the app) | 4 (4.9%) | 3 (4.7%) | 1 (5.9%) | 1.000^ |
|  | 1.2 | The app has a feature that allows the user to switch between daily and weekly medication schedule displays | 3 (3.7%) | 1 (1.6%) | 2 (11.8%) | 0.110^ |
|  | 1.3 | The app has a feature that allows the user to schedule medication-taking on alternate days (e.g. Pill A on Monday, Wednesday, Friday; Pill B on Tuesday, Thursday, Saturday etc.) | 24 (29.6%) | 17 (26.6%) | 7 (41.2%) | 0.241 |
|  | 1.4 | The app has a feature to enter the purpose of the medication | 22 (27.2%) | 16 (25.0%) | 6 (35.3%) | 0.540^ |
|  | 1.5 | The app has a feature that allows the user to enter special instructions for medication (e.g. taken before food) | 33 (40.7%) | 27 (42.2%) | 6 (35.3%) | 0.607^ |
|  | 1.6 | The app has a feature that allows the user to organize “take as needed” medications in a separate section from medicines with a fixed regimen | 6 (7.4%) | 1 (1.6%) | 5 (29.4%) | 0.001^* |
|  | 1.7 | The app has a feature that allows the user to enter/log at least 4 different medications at any given time | 56 (69.1%) | 42 (65.6%) | 14 (82.4%) | 0.184^ |
|  | 1.8 | The app has a variety of dosage input options (e.g. subcutaneous insulin for diabetes, oral medications) | 66 (81.5%) | 49 (76.6%) | 17 (100.0%) | 0.032^* |
|  | 1.9 | The app has a feature that allows the user to document allergies (i.e. via prompts/greyed out instructions or a separate tab) | 4 (4.9%) | 2 (3.1%) | 2 (11.8%) | 0.192 |
|  | 1.10 | The app has a feature that allows the user to sync medication-taking schedule with the phone calendar | 2 (2.5%) | 2 (3.1%) | 0 (0.0%) | 1 |
| Monitoring and Adherence | 2.1 | The app has a feature that allows the user to record the fraction of an actual pill or volume of a liquid medication prescribed (i.e. ½ pill or 5 ml of a syrup) to be recorded. | 45 (55.6%) | 35 (54.7%) | 10 (58.8%) | 0.76 |
|  | 2.2 | The app has a feature that allows users to document medication-intake | 57 (70.4%) | 41 (64.1%) | 16 (94.1%) | 0.016* |
|  | 2.3 | The app has a feature that allows users to record notes on any medication event (i.e. a “note/comment” section at the logging page or a as a separate tab) | 35 (43.2%) | 25 (39.1%) | 10 (58.8%) | 0.144 |
|  | 2.4 | The app has a feature that allows users to document medication side-effects (i.e. via prompts/greyed-out instructions or a separate tab) | 4 (4.9%) | 2 (3.1%) | 2 (11.8%) | 0.192^ |
|  | 2.5 | The app has a feature that assesses medication adherence by comparing planned and actual medication taking (E.g. the app generates weekly percentage of adherence or has a visual display). | 17 (21.0%) | 12 (18.8%) | 5 (29.4%) | 0.334^ |
| Information provision | 3.1 | The app has a feature that provides users with information about the prescribed medication | 6 (7.4%) | 4 (6.3%) | 2 (11.8%) | 0.601^ |
|  | 3.2 | The app has a feature that provides users with resources (in-app or external link) to access information about the prescribed medication | 4 (4.9%) | 3 (4.7%) | 1 (5.9%) | 1.000^ |
| Complementary medicines | 4.1 | The app has a feature that asks users about the use of complementary medicines | 0 (0.0%) | 0 (0.0%) | 0 (0.0%) | 1.000^ |
|  | 4.2 | The app has a feature that flags possible contraindications with the use of complementary medicines | 0 (0.0%) | 0 (0.0%) | 0 (0.0%) | 1.000^ |
| Reminders | 5.1 | The app has a feature that allows users to set up reminders for taking medications | 47 (58.0%) | 37 (57.8%) | 10 (58.8%) | 0.94 |
|  | 5.2 | The app has a feature that allows users to set up reminders to refill prescriptions | 9 (11.1%) | 3 (4.7%) | 6 (35.3%) | 0.002^* |
| Motivation | 6.1 | The app has a feature that provides statements to motivate users about the importance of medication adherence | 3 (3.7%) | 2 (3.1%) | 1 (5.9%) | 0.512^ |
|  | 6.2 | The app has a feature that provides encouragement when medication is taken on schedule (i.e. encouraging messages, “badges or awards”) | 3 (3.7%) | 1 (1.6%) | 2 (11.8%) | 0.110^ |
| Caregiver’s involvement | 7.1 | The app has a feature that allows users to sync medication-taking schedule with caregiver’s phone | 5 (6.2%) | 1 (1.6%) | 4 (23.5%) | 0.006^* |
|  | 7.2 | The app has a feature that supports multiple user profiles (e.g. For family members or carers) | 11 (13.6%) | 5 (7.8%) | 6 (35.3%) | 0.009^* |
| Communication with healthcare provider | 8.1 | The app has a feature that allows users to contact a healthcare provider regarding queries on medication | 7 (8.6%) | 4 (6.3%) | 3 (17.6%) | 0.157^ |
| Communication with health system | 9.1 | The app has a feature that supports data export | 44 (54.3%) | 29 (45.3%) | 15 (88.2%) | 0.002^ |

^Two-tailed p-value calculated using Fisher’s-exact test as the expected count is less than 5 in at least a group

*Statistical significance P<0.05 in the comparison of app features between Android apps with <100,000k downloads and ≥100,000k downloads.
